# Low cardiorespiratory fitness is associated with elevated intraocular pressure among apparently healthy adults

**DOI:** 10.1371/journal.pone.0302624

**Published:** 2024-04-29

**Authors:** Nir Stanescu, Lioz Steinbuch, Amit Segev, Natalya Kovalyuk, Shlomo Segev, Elad Maor, Fani Segev

**Affiliations:** 1 Department of Ophthalmology, Samson Assuta Ashdod Medical Center, Ashdod, Faculty of Medicine, Ben-Gurion University of the Negev, Beersheba, Israel; 2 Chaim Sheba Medical Center, Tel Hashomer, Faculty of Medicine, Tel Aviv University, Tel Aviv, Israel; 3 Division of Cardiology, Chaim Sheba Medical Center, Tel Hashomer, Faculty of Medicine, Tel Aviv University, Tel Aviv, Israel; 4 The Institute of Medical Screening, Chaim Sheba Medical Center, Tel Hashomer, Faculty of Medicine, Tel Aviv University, Tel Aviv, Israel; Alexandria University Faculty of Medicine, EGYPT

## Abstract

**Purpose:**

To evaluate the association of cardiorespiratory fitness with elevated intraocular pressure (IOP) in healthy adults.

**Methods:**

In this cross-sectional study, we evaluated 17,990 asymptomatic self-referred adults free of diabetes or cardiovascular disease who were screened in a preventive healthcare setting. All subjects underwent measurement of IOP and completed a maximal exercise stress test according to the Bruce protocol. Fitness was categorized into age and sex-specific quintiles according to the treadmill time and dichotomized to low (lowest quintile) and non-low fitness groups. Elevated IOP was defined as ≥ 21 mmHg.

**Results:**

Median age was 45 (IQR 39–52) years and 12,073 (67%) were men. There were 3,351 (19%) subjects in the low fitness group. Median IOP was 14 mmHg (IQR 12–16) with elevated IOP documented in 188 (1%) subjects. Univariate binary logistic regression model demonstrated that compared with non-low fitness group, subjects in the low fitness group were 2.2 times more likely to have elevated IOP (95% CI 1.598–2.95, p<0.001). Multivariate binary logistic regression with adjustment to known cardiovascular risk factors (age, sex, hypertension, smoking, overweight, regular physical activity, low HDL cholesterol, high triglycerides, and fasting glucose levels) successfully demonstrated that lower fitness was independently and significantly associated with a 90% increased likelihood of elevated IOP (95% CI 1.37–2.61, p<0.001). Subgroup analysis revealed that the association was more pronounced among women compared with men (OR 3.8 vs. 1.6, p for interaction = 0.069).

**Conclusions:**

Low cardiorespiratory fitness is independently associated with increased IOP among apparently healthy adults.

## Introduction

The pressure of fluids inside the eye is known as intraocular pressure (IOP). In physiological conditions, it is determined by the delicate balance between ocular structures that produce aqueous humor (e.g., ciliary body) and structures that eliminate it (e.g., trabecular meshwork). Large population-based studies have found that the mean IOP is 15–16 mmHg, and the upper limit of normal IOP was traditionally defined as two standard deviations above it, that is, 21 mmHg [1]. Chronically elevated IOP has been associated with retinal ganglion cell death, and was hypothesized as one of the possible pathological mechanisms for the development of glaucoma- a disease that causes an irreversible damage to the optic nerve, and reduces vision-related quality of life. Although recent studies have revealed high interpersonal variability between IOP glaucoma, lowering the IOP is still the only available treatment modality [1, 2].

The relationship between physical exercise and intraocular pressure (IOP) goes as far back as the 1960’s [3]. A large body of evidence suggests that acute exercise such as treadmill or cycling, causes an acute rise in IOP at volitional exhaustion [4] followed by a decrease in IOP in the immediate post exercise period [5–8]. An early study by Sargent et al., which included 32 adult subjects in total, investigated the effects of a 6-month training program and its effects on IOP for an intervention group compared to a control group. They found a significantly lower IOP after the training program. However, there was not a statistically significant difference between the intervention and control group [9].

Cardiorespiratory fitness (CRF) has a well-established association with cardiovascular outcomes and all-cause mortality [10–12]. Recently, Meier et al. demonstrated that low CRF was associated with incident glaucoma, suggesting that meeting physical activity guidelines or being fit reduces the risk of developing glaucoma [13]. However, robust clinical data on the association of CRF with elevated IOP is lacking and more data in the field is warranted. Therefore, the aim of this study was to investigate the relationship between CRF and IOP in a large cohort of healthy self-referred adults in preventive healthcare settings.

## Methods

### Study population

We described the study population in previous work done by our group [14]. Briefly, participants were self-referred healthy adults that underwent a comprehensive screening evaluation as part of executive health insurance programs or as private individuals in a single medical center. Evaluation included a physical examination, an ophthalmic examination, health questionnaire, blood analysis, blood pressure measurements, and an exercise stress testing (EST) using the Bruce protocol [15].

The institutional review board of Sheba medical center approved the study under the condition of the deidentification of the participants and the strict maintenance of their anonymity (approval number SMC-8995-11). Therefore, no individual consent was obtained. The study adhered to the tenets of the declaration of Helsinki.

### Inclusion and exclusion criteria

On August 20^th^, 2022 we first accessed our database that included 28,695 deidentified participants who were examined between August 8^th^, 2000 and May 4^th^ 2017. Only participants who completed an EST and had a documented measurement of IOP were included. Exclusion criteria included missing EST data, missing IOP measurements, diabetes mellitus or cardiovascular disease (ischemic heart disease, cerebrovascular stroke, or atrial fibrillation) at baseline.

### Study design

Participants underwent a treadmill EST according to the Bruce protocol [15]. Each test was supervised by a board-certified cardiologist. Participants were encouraged to reach their age specific maximal heart rate, but the test was interrupted earlier in cases of exhaustion, angina or any other medical condition that did not allow the continuation of the test. Exercise-test time was used to estimate oxygen consumption for the aerobic exercise and was expressed in metabolic equivalents (METs). This conversion was done by precise regression equations recommended by the American College of Sports Medicine [16–19]. Using this protocol exercise test time is highly correlated to directly measured maximal oxygen uptake (i.e., CRF) [20]. Definition of CRF groups in this specific population was previously described [21]. First, we computed age-specific and sex-specific distribution of treadmill duration for the following age groups: 40–49, 50–59, 60–69 and 70–79 years. Then, each sex-specific and age-specific subgroup was divided into 5 quintiles of CRF based on treadmill duration time. We combined these quintiles of CRF measures into two mutually exclusive groups: low CRF (Q1, n = 3,354 [19%]) and non-low CRF, (Q2-Q5, n = 14,636 [81%]). In addition, other well-known cardiovascular risk factors were obtained for each participant, these included age, sex, hypertension overweight, smoking, self-reported regular physical activity, HDL-cholesterol level, fasting glucose level and triglycerides level. All continuous variables except for age were dichotomized in the following manner: overweight was defined as BMI > 25 kg/m^2^, low HDL-cholesterol level was defined as HDL < 50 mg/dL for women and HDL < 40 mg/dL for men, impaired fasting glucose was defined as a glucose level > 100 mg/dL, and hypertriglyceridemia was defined as triglycerides > 150 mg/dL. Participants were considered to have arterial hypertension if the diagnosis was documented in their electronic medical records, if they were on long‐term antihypertensive drug therapy, or if they had 2 separate measurements of either a systolic BP > 140 mm Hg and/or a diastolic BP > 90 mm Hg.

### Intraocular pressure measurement

Each participant underwent an IOP measurement of both eyes. The measurement was performed before the EST by a board-certified ophthalmologist, using a non-contact tonometer (HNT-7000, Huvitz tonometer, Gunpo, Korea). Results were recorded in ‘mmHg’ units. In cases where the IOP measurement exceeded or equaled 21 mmHg, we conducted a second measurement of IOP using a Goldman applanation tonometer and recorded the second measurement in the participant’s medical record.

### Study endpoint

The main outcome measure of the study was an elevated IOP. An elevated IOP value was defined as ≥21 mmHg in at least one of the subject’s two eyes, as the current consensus [1, 22].

### Statistical analysis

Continuous parameters of the study groups were compared using the student’s t-test. For comparison of categorical data, we used the χ^2^ test. First, univariate binary logistic regression was used to determine the unadjusted odds ratio (OR) for elevated IOP of different CRF quintiles with the highest quintile used as reference. Univariate binary logistic regression was also used to determine the unadjusted OR for elevated IOP of other known cardiovascular risk factors. Then, a multivariate binary logistic regression with adjustment to the same cardiovascular risk factors was used to determine the adjusted OR for elevated IOP among low CRF group compared with non-low CRF group (quintiles Q2-Q5). The quality of the binary logistic regression adjustment model was assessed using Hosmer-Lemeshow test for goodness of fit. Using the same model, adjusted OR was evaluated in the following sub-groups: men vs. women, overweight vs. normal weight, smoking vs. non-smoking, physically active vs. non-active, low HDL-cholesterol level vs. non-low HDL-cholesterol level, impaired fasting glucose vs. normal fasting glucose and hypertriglyceridemia vs. normal triglycerides. For each subgroup analysis, interaction analysis was evaluated as well. All statistical analyses were performed using SPSS software (SPSS version 21, IBM, Chicago, Illinois). An association was considered statistically significant for a 2-sided P value of < 0.05.

## Results

**Fig 1:**
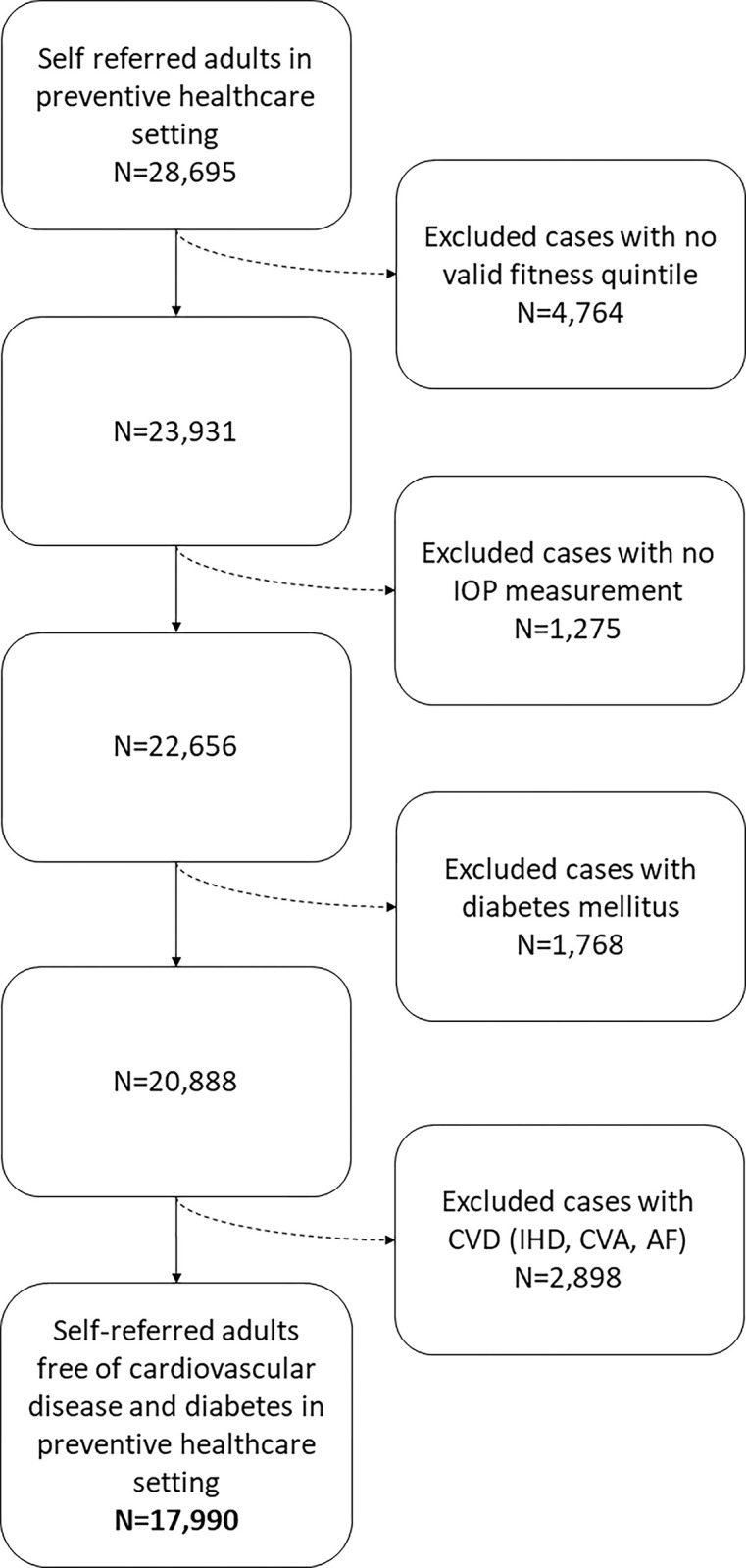
Participants selection flow chart. A participant flow chart describing the selection process for our study population. CVD = cardiovascular disease; IHD = ischemic heart disease; CVA = cerebrovascular accident; AF = atrial fibrillation.

The final study population comprised 17,990 individuals, of whom 12,073 (67%) were men (Fig 1). Mean age was 46±10 years (median 45, IQR 39–52). Baseline clinical and laboratory characteristics of study subjects by the CRF level groups are presented in Table 1.

**Table 1:** Baseline clinical characteristics of the study population by cardiorespiratory fitness group.

Characteristic	All Subjects	Low CRF	Non-Low CRF	P Value
Age±SD [years]	46±10	46±10	46±9	0.207
Females N (%)	5917 (33)	1160 (35)	4757 (33)	0.019
BMI±SD [kg/m^2^]	25 ±5	27±5	25±4	<0.001
Overweight N (%)	9230 (51)	2132 (64)	7098 (48)	<0.001
Blood Pressure±SD[mmHg]	119/75±20/13	123/78±21/13	118/75±19/13	<0.001
Hypertension N (%)	2745 (15)	675 (20)	2070 (14)	<0.001
Active smoking N (%)	3459 (19)	644 (19)	2815 (19)	0.999
Physically Active N (%)	11482 (63)	1458 (43)	10024 (68)	<0.001
HDL±SD [mg/dL]	49±13	47±12	49±13	<0.001
Low HDL N (%)	5565 (31)	1286 (38)	4279 (29)	<0.001
Triglycerides±SD [mg/dL]	121±69	137±78	117±67	<0.001
Hypertriglyceridemia N (%)	4285 (23.8)	1103 (33)	3182 (22)	<0.001
Fasting Glucose±SD [mg/dL]	88±11	89±11	87±10	<0.001
IFG N (%)	1684 (9)	421 (13)	1263 (9)	<0.001
IOP±SD [mmHg]	14±2.5	14.2±2.7	13.9±2.45	<0.001
Elevated IOP N (%)	188 (1)	62 (1.9)	126 (0.9)	<0.001

CRF = cardiorespiratory fitness; BMI = body mass index; HDL = high density lipoprotein; IFG = impaired fasting glucose; IOP = intra ocular pressure.

Notably, subjects in the non-low CRF group had lower IOP, lower BMI, lower blood pressure, higher physical-activity rate, higher HDL-cholesterol levels, lower triglycerides levels and lower fasting glucose levels (Table 1).

**Fig 2:**
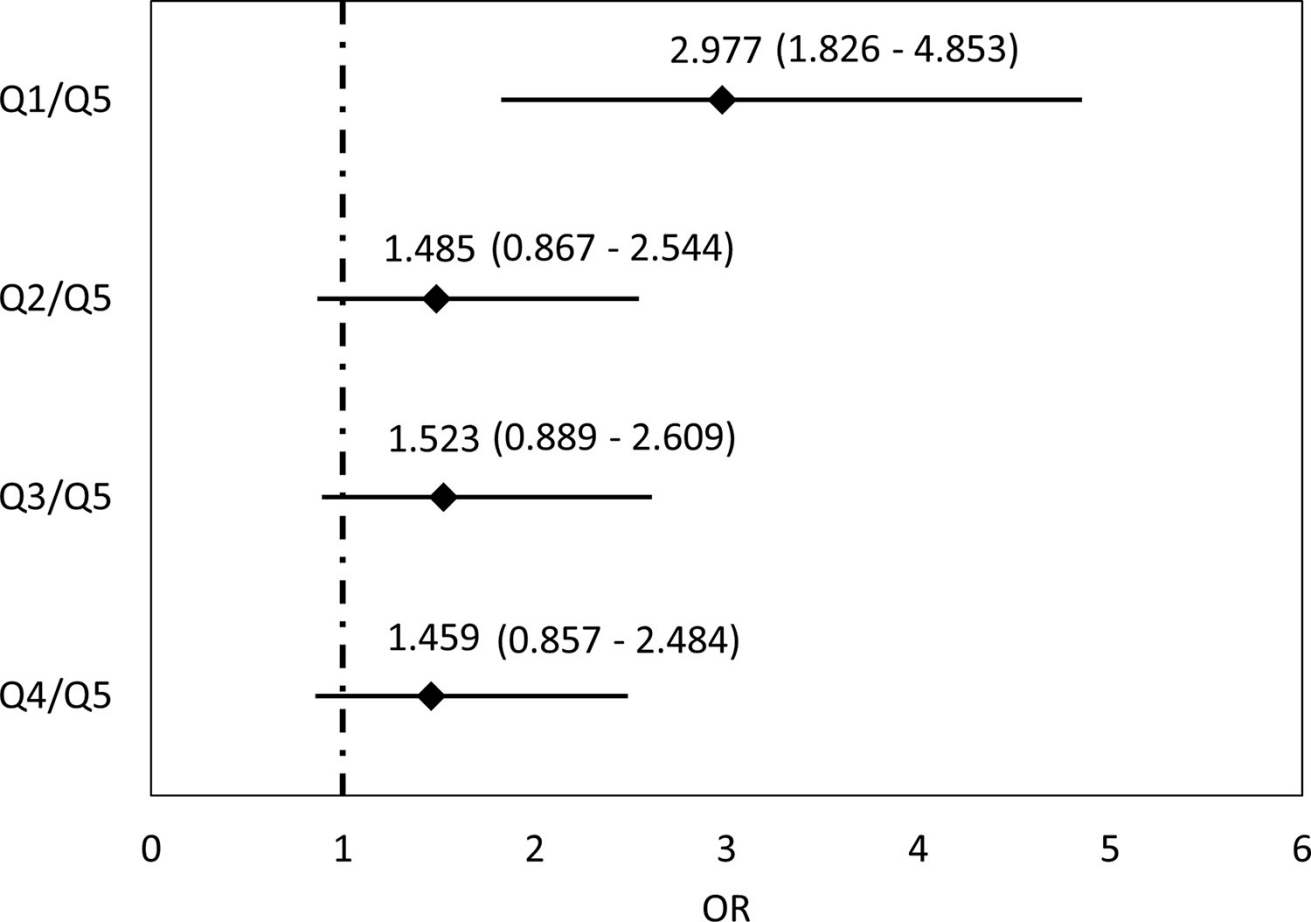
Unadjusted OR of elevated IOP by fitness group. The difference in OR between the first quintile and the rest of the quintiles can be seen clearly. This finding has led to the separation and definition of quintile 1 as the low CRF group and quintiles 2–5 as non-low CRF group. OR = odds ratio; IOP = intra ocular pressure; CRF = cardiorespiratory fitness.

The mean value and standard deviation of IOP for the entire research population was 14±3 mmHg. Distribution of IOPs were close to normal, with only 135 cases of IOP lower than 8 mmHg and 188 cases of IOP ≥ 21 mmHg. There were 62 (1.9%) subjects with elevated IOP in the low CRF group vs. 126 (0.9%) subjects with elevated IOP in the non-low CRF group (p < .001). Univariate binary logistic regression is graphically presented in Fig 2. Overall, the model showed that compared with non-low CRF group, subjects in the low CRF group were 2.2 more likely to have elevated IOP (95% CI 1.598–2.95, p < 0.001). In addition to low CRF, other cardiovascular risk factors were also associated with increased likelihood of elevated IOP. Multivariate binary logistic regression with adjustment to the same cardiovascular risk factors demonstrated that low CRF was independently and significantly associated with a 90% increased likelihood of elevated IOP (95% CI 1.37–2.61, p<0.001). Additional factors in the multivariate model independently associated with increased odds ratio for elevated IOP were sex, overweight and low HDL-cholesterol. The Hosmer-Lemeshow goodness of fit test for the multivariate model yielded a chi square statistic of 13.8 (p = 0.086), indicating a good fit of the model to the data. Results of the univariate predictors of elevated IOP and multivariate model for predicting elevated IOP are summarized in Table 2.

**Table 2:** Univariate and multivariate binary logistic regression models, using the same variables for the prediction of elevated IOP.

Variable Name	Univariate		Multivariate	
	OR (95% CI)	P-Value	OR (95% CI)	P-Value
Age	1.06 (1.046–1.074)	<0.001	1.055 (1.04–1.07)	<0.001
Female Sex	0.4 (0.272–0.588)	<0.001	0.463 (0.311–0.692)	<0.001
Overweight	2.314 (1.688–3.173)	<0.001	1.506 (1.076–2.108)	0.017
Active smoking	0.583 (0.376–0.903)	0.016	0.636 (0.408–0.992)	0.046
Physically Active	0.872 (0.65–1.17)	0.361	0.9 (0.661–1.226)	0.505
IFG	2.161 (1.486–3.144)	<0.001	1.185 (0.801–1.753)	0.397
HTN	2.088 (1.51–2.887)	<0.001	1.019 (0.718–1.447)	0.916
Low HDL	1.488 (1.11–1.996)	0.008	1.445 (1.057–1.976)	0.021
Hypertriglyceridemia	1.397 (1.022–1.91)	0.036	0.929 (0.664–1.299)	0.666
Low CRF	2.171 (1.598–2.95)	<0.001	1.897 (1.377–2.613)	<0.001

OR = odds ratio; IOP = intra ocular pressure; IFG = impaired fasting glucose; HTN = hypertension; HDL = high density lipoprotein; CRF = cardiorespiratory fitness.

**Fig 3:**
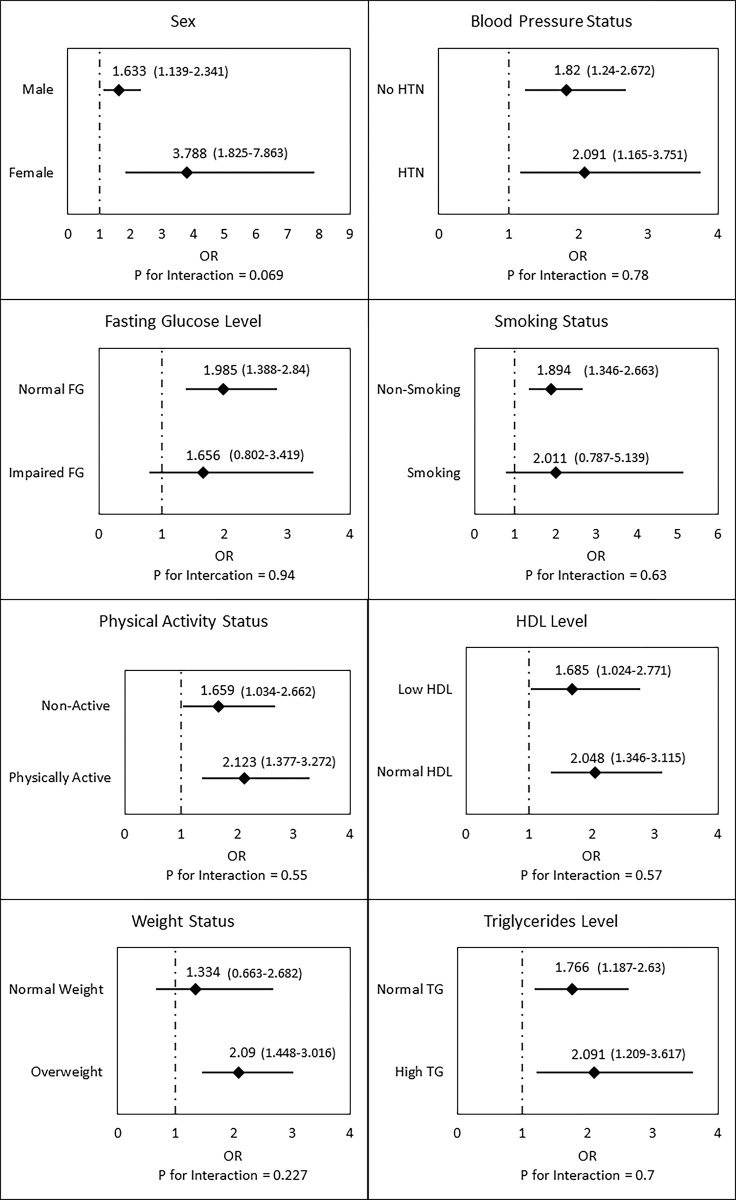
Adjusted OR for elevated IOP by fitness. Forest plots for the subgroup analysis of the association between elevated IOP and low CRF. It is demonstrated that the association is more pronounced in women (OR of 3.79) and in overweight (OR of 2.09). OR = odds ratio; IOP = intra ocular pressure; CRF = cardiorespiratory fitness.

Subgroup analysis demonstrated consistent results in all subgroups evaluated, with a trend towards a stronger association between low CRF and elevated IOP among women vs. men (OR 3.79, 95% CI 1.82–7.86, p < 0.001 for women vs. OR 1.63, 95% CI 1.14–2.34, p < 0.001; p for interaction = 0.069) and among overweight vs. non-overweight subjects (OR 2.1, 95% CI 1.45–3.02, p < 0.001 for overweight subjects vs. OR 1.33, 95% CI 0.66–2.68, p < 0.001; p for interaction = 0.227) (Fig 3).

## Discussion

The main finding of this study is that objectively measured low CRF is associated with an increased likelihood of elevated IOP among apparently healthy middle-aged subjects. The association was significant in a multivariate model and among all subgroups evaluated suggesting an independent association. Moreover, the association between low CRF and elevated IOP was more pronounced among females and among overweight subjects.

The latest edition of the preferred practice patterns of the American academy of ophthalmology (AAO) for open angle glaucoma states that no absolute value of IOP should serve as a cutoff value for glaucoma diagnosis. This is due to recent population-based studies that have demonstrated a highly variable proportion of patients with elevated IOP who developed glaucoma [2]. Consequently, our results cannot be used to infer the risk of developing glaucoma. Nevertheless, Meier et al. demonstrated that individuals with low CRF calculated by a treadmill exercise stress test, and with low self-reported physical activity per week, have a higher risk of incident glaucoma [13]. The study was a retrospective cohort study involving 9519 participants with a mean age of 50 years and a mean follow-up time of 6 years. Curiously, they did not provide any information regarding IOP measurements.

An interesting prospective study that supports our results is the study of Sargent et al. This was a small (N = 32) interventional randomized controlled trial that was published in 1981. All participants had IOPs of greater than 18mmHg who were divided in two groups: An intervention group (N = 18), that was prescribed a 6-months supervised training program, and a control group (N = 14). They managed to demonstrate a statistically significant increase in the intervention group CRF score and an associated reduction in IOP in each eye. However, the control group also showed a significant reduction in IOP in the right only. Furthermore, unpaired t-test did not find a statistically significant difference between the reduction in IOP between the groups; and linear regression did not find a statistically significant relationship between CRF and IOP. They attributed their results to a small sample size and to artifacts related to their method of IOP measurements [9].

Vera et al. investigated IOP dynamics following strenuous effort on a treadmill in both physically trained and untrained military personnel. Both groups exhibited an acute increase in IOP at maximal effort, followed by a reduction in IOP at 5 minutes of rest. The group with the higher fitness level displayed a more stable IOP curve, attenuating the IOP spike at the peak of the effort. They suggested that high-intensity effort (i.e., to exhaustion) should be discouraged in individuals with glaucoma [4].

Noteworthy is the study of Era et al. that did not find any statistically significant differences in IOP between elderly athletes and controls performing bicycle ergometry. It included both male and female athletes (selected randomly from veteran members of Finnish sports organizations) and controls. Overall, there were 161 participants with a mean age of 74 years. They calculated the mean IOP in each group before conducting the bicycle ergometry and did not find any statistically significant difference between the presumably fit participants and those that were not [23].

A large-scale study by Qureshi showed that women have significantly higher IOP than men. This study included 7695 healthy volunteers from Rawalpindi, Pakistan. Participants in the study were divided into 7 age groups (10 to 71+). There was an age dependent increase in IOP in both sexes until the seventh decade (age group 61–70). Starting at the fifth decade, the mean IOP in the female groups was significantly higher than their male counterparts. Moreover, the IOP distribution curve in the female group was much more skewed to the right than the corresponding male curve, meaning that females showed a higher tendency towards higher IOP measurements [24]. However, higher IOP in men was found in large population-based studies in Korea and in Japan [25, 26]. A possible explanation to these contradictory findings could be found in our results. It is possible that an interacting factor such as CRF influenced the relationship between sex and IOP.

As observed in other studies, BMI is positively correlated with IOP [26–28]. We demonstrated that overweight individuals (with BMI ≥ 25 kg/m^2^) had twice as much odds of having elevated IOP than their lower BMI counterparts. Our findings are supported by a large scale study from Israel by Cohen et al. who showed a statistically significant positive association between BMI and IOP ≥ 18 mmHg [28]. Not only that our results are concordant with their findings, but we have also shown that such an association is still valid for IOP ≥ 21 mmHg.

The current consensus in the ophthalmic community is that smoking has a detrimental effect on the visual system including the risk for elevated IOP and incident glaucoma [29, 30]. Surprisingly, in this large-scale study we have found that holding all other predictor variables constant, the odds of having IOP ≥ 21 mmHg decreased by 36% (95% CI 1%-60%, p = 0.046) for smokers. Hopefully, future studies would shed more light on the relationship between smoking and IOP.

Our study has several limitations. First, the study population consists only of self-referred healthy adults who completed an exercise stress test and an IOP measurement, and composed mainly of relatively young (i.e., mean age 46 years) Caucasian men and women from a high socioeconomic background which may not be representative of the general population, may potentially limit the generalizability of the results and might induce a bias towards lower IOP measurements [2, 31]. Second, we did not record corneal pachymetry. Third, the IOP measurement was done only once, and it might not be a representative measurement of the average long-term IOP of the participants and might had been influenced by temporaneous factors. Fourth, it is a cross-sectional study with no follow-up period. Fifth, the covariates sex and overweight status did not reach the predetermined statistical significance value of 0.05 for their interaction with the association between CRF and IOP, however, we still chose to point them out for their clinical relevance.

In conclusion, this large-scale population-based study demonstrated that individuals with low CRF are twice as likely to have elevated IOP; and that this relationship is further augmented in women and in overweight individuals. Future studies should concentrate on changes in IOP over time in different CRF groups, and the rates of incident glaucoma in each group.

## Supporting information

S1 Checklist Human participants research checklist. (DOCX)
